# Mid-infrared ultrafast laser pulses induced third harmonic generation in nitrogen molecules on an excited state

**DOI:** 10.1038/srep16006

**Published:** 2015-11-02

**Authors:** Hongqiang Xie, Guihua Li, Jinping Yao, Wei Chu, Ziting Li, Bin Zeng, Zhanshan Wang, Ya Cheng

**Affiliations:** 1 State Key Laboratory of High Field Laser Physics, Shanghai Institute of Optics and Fine Mechanics, Chinese Academy of Sciences, Shanghai 201800, China; 2 University of Chinese Academy of Sciences, Beijing 100049, China; 3 School of Physics Science and Engineering, Tongji University, Shanghai 200092, China

## Abstract

We report on generation of third harmonic from nitrogen molecules on the excited state with a weak driver laser pulse at a mid-infrared wavelength. The excited nitrogen molecules are generated using a circularly polarized intense femtosecond pulse which produces energetic electrons by photoionization to realize collisional excitation of nitrogen molecules. Furthermore, since the third harmonic is generated using a pump-probe scheme, it enables investigation of the excited-state dynamics of nitrogen molecules produced under different conditions. We also perform a comparative investigation in excited argon atoms, revealing different decay dynamics of the molecules and atoms from the excited states in femtosecond laser induced filaments.

Recently, free-space air lasing actions induced by intense ultrafast laser pulses have attracted growing attention. On the one hand, the capability of remotely generating intense, narrow-bandwidth, coherent emissions in air opens new horizon for remote sensing^1^,^2^,^3^,^4^,^5^,^6^,^7^,^8^. On the other hand, as a novel nonlinear optical process, lasing actions in air typically involve complex interplay between various effects in molecules such as dissociation, excitation and ionization of molecules, alignment of neutral and ionized molecules. Therefore, air lasers offer an ideal tool for investigating strong-field molecular physics and ultrafast nonlinear spectroscopy^9^,^10^. Furthermore, both ground and excited electronic states are involved in the establishment of population inversion responsible to the generation of air lasers. Therefore, efficient generation of air lasers requires preparing the molecules on the excited states with an intense laser field^5^,^7^,^8^. Meanwhile, it is equally important to understand the decay dynamics of the excited states for the molecules which serve as the gain medium, which has not been quantitatively examined in a systematic way.

In the previous investigations, we have demonstrated that air lasers generated with a pump-probe scheme have the ability to probe the ultrafast dynamics of population difference between the upper and lower energy states as lasing typically requires population inversion^7^,^9^,^11^. Therefore, the temporal dynamics of air laser signals obtained using the pump-probe scheme cannot provide direct information on the decay dynamics of molecules on excited states. Although the temporal information of the decay of the excited states may be obtained using a time resolved detection of spontaneous fluorescence emission from the excited-state molecules^12^, it requires a high speed streak camera which is not always accessible. Here, we achieve a temporal resolution of ~16.7 fs in the investigation of the decay dynamics of excited nitrogen molecules produced in intense laser fields. The strategy we choose is to generate third harmonic (TH) signal in the excited-state molecules because of the higher third-order nonlinear susceptibility on the excited state^13^,^14^,^15^,^16^,^17^. Therefore, the TH signal can serve as a probe of the ultrafast dynamics of excited states if the excitation of the atoms or molecules can be accomplished using ultrafast pump laser pulses. The demonstrated approach can be employed as a useful tool to investigate the ultrafast dynamics of excited molecules and atoms produced by femtosecond laser pulses, which is of importance for applications including remote atmospheric sensing, air lasing, and strong field molecular physics.

## Results and Discussions

**Figure 1:**
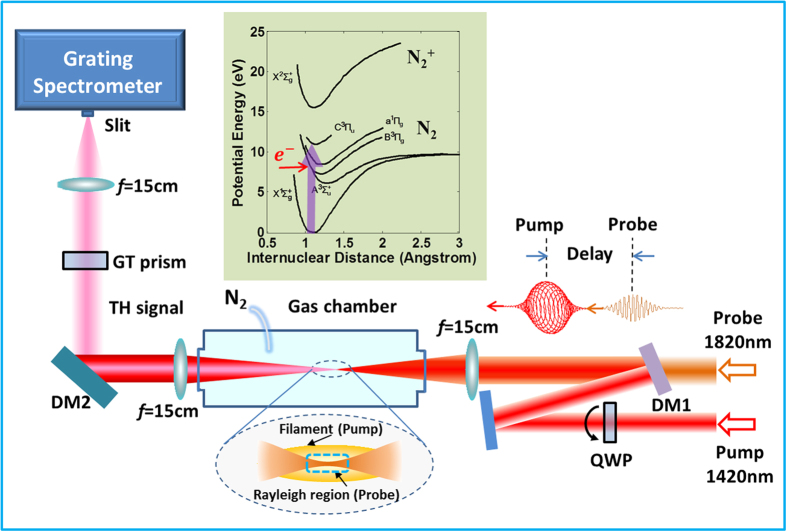
Schematic diagram of experimental setup. Inset: potential energy curves of the electronic states of nitrogen molecules.

Our experimental setup is illustrated in Fig. 1. The experimental details are provided in **Methods**. Briefly speaking, in the experiment, a signal beam at the wavelength of 1420 nm from an optical parametric amplifier (OPA) was used as the pump to prepare the excited states, while an idler beam at the wavelength of 1820 nm served as the probe to generate TH from excited-states atoms or molecules. The energy of the pump pulse was fixed at ~630 μJ, which is the maximum output pulse energy from the OPA for generating the highest population on the excited states. In contrast, the probe laser with the pulse energy of only 2 μJ is much weaker. We have observed that the minimum energy of the probe pulse required for third harmonic generation (THG) is ~50 μJ under our experimental conditions, which is much higher than that used in the pump-probe experiment (i.e., 2 μJ). Thus, at such low pulse energy, the TH signal from the nitrogen molecules on the ground state can be eliminated. Namely, the TH signal is purely from the nitrogen molecules on the excited state.

**Figure 2:**
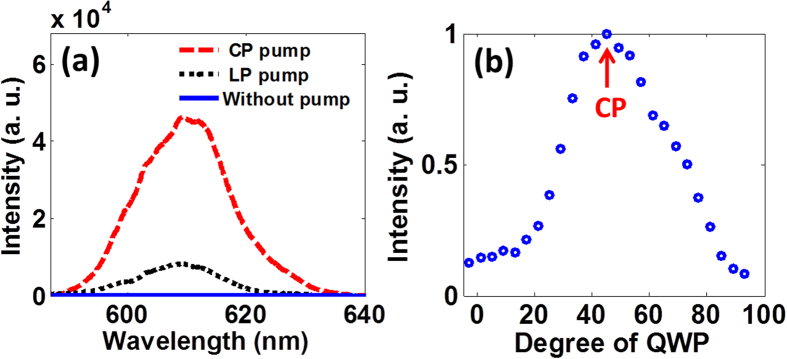
(**a**) TH spectra generated by an 1820 nm, 2 μJ probe pulse in the cases with CP pump, LP pump and without pump. The time delay of the pump and the probe is ~1.3 ps. (**b**) The intensity of TH signal as a function of the polarization of the pump laser.

Figure 2(a) shows typical TH spectra of the probe pulse generated in nitrogen gas at 1000 mbar. Clearly, the probe laser alone with the pulse energy of 2 μJ cannot generate detectable TH signal, as shown by the blue solid curve in Fig. 2(a). However, when an intense pump pulse is focused into the gas chamber at ~1.3 ps ahead of the probe pulse, the strong TH signal can be observed. It can be seen that the center wavelength of TH signal of the probe pulse is 607.2 nm and the bandwidth (FWHM) is ~18 nm. Interestingly, TH signal generated with the circularly-polarized (CP) pump is about 5 times stronger than that generated with the linearly-polarized (LP) pump. It is worth mentioning that the TH signal has almost the same intensity when the pump pulse has either a left or a right circular polarization. To gain further insight into the dependence of TH signal on pump laser polarization presented in Fig. 2(a), we further measured the intensity of TH signal as a function of the rotation angle ϕ of the quarter wave plate (QWP) inserted in the pump beam. As shown in the Fig. 2(b), TH signal reaches its maximum in the case of circular polarization (i.e., ϕ = 45°) and gradually decreases when the pump laser polarization deviates from circular polarization.

Based on the above observations, we first attempt to identify whether the TH generated with the pump-probe scheme originates from the formation of excited states of nitrogen molecules. There are two possible mechanisms responsible for the enhancement of TH signal when both the pump and probe laser pulses are focused into the nitrogen gas. First, the plasma generated by the pump pulse could give rise to either an improved phase matching^18^,^19^ or an enhanced nonlinear polarizability^20^. In both cases, the enhancement of TH signal critically depends on the plasma density. It is well known that atoms or molecules can be more easily ionized in the LP laser field than in the CP laser field^21^,^22^. Thus, if the enhanced THG would be due to the plasma effect, the enhancement will be stronger with a LP pump laser. However, in reality, our experimental results show that TH signal can be more efficiently enhanced with the CP pump pulses. Thus, the plasma effect would not play the dominant role on the enhanced TH signal.

Another possibility is that the efficient THG is due to the enhanced nonlinear polarizability of the nitrogen molecules on the excited states^13^,^14^,^15^,^16^,^17^. As suggested in previous work, the nitrogen molecules can be populated to the excited electronic states by CP pump pulses at the 800 nm^8^,^11^ and LP pump pulses at the 4 μm^23^ via collision between hot electrons and neutral nitrogen molecules, which gives rise to generation of free-space nitrogen lasers. Cross sections of electron collisions with nitrogen molecules critically depend on the electron temperature (i.e., the average kinetic energy of electron)^24^. It is known that in the classical model of strong field ionization (i.e., the simple man’s picture)^25^, the peak energy of tunnel ionized electrons will gradually increases from zero to ~2 U_p_ (U_p_ the pondermotive energy) by increasing the ellipticity of the polarization of the pump pulses, producing more nitrogen molecules on the excited states. Therefore, THG is more efficient when the CP pump laser is employed.

**Figure 3:**
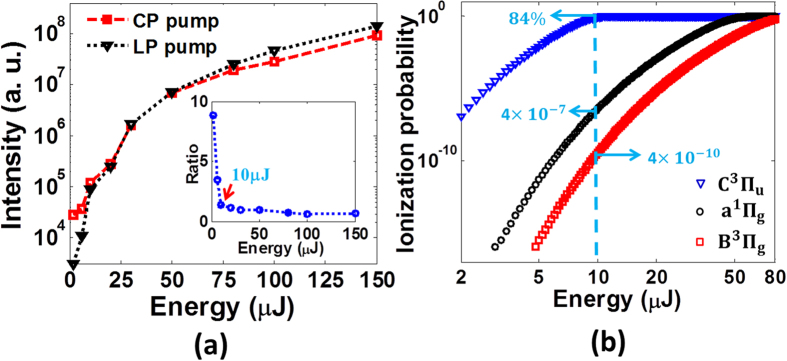
(**a**) The intensity of TH signal as a function of the probe energy for the case of CP pump and LP pump. Inset: The ratio of TH signal generated with the CP pump and that generated with the LP pump as a function of the probe energy. (**b**) The calculated ionization probabilities of three excited states of nitrogen molecules as a function of the probe energy.

Figure 3(a) presents that with the increase of the probe energy, TH signals firstly increase rapidly and then tend towards saturation in both cases of CP pump and LP pump. Moreover, the ratio of TH signal generated with the CP pump and that generated with the LP pump shows a sharp decrease below the 10 μJ followed by a slow decay from 10 μJ to 100 μJ, as illustrated in the inset of Fig. 3(a). A turning point appears at the probe energy of 10 μJ, as indicated by a red arrow. Below the turning point, THG is in favor of the CP pump, whereas LP pump is more efficient for THG above the turning point. The reason is that, with the increase of the probe energy, population on the excited states will be depleted by photoionization, thus the TH signal mainly originates from the molecules on the ground state. In such a case, the plasma effect of the pump laser becomes more important for THG, and thus the TH signal at the higher probe energy becomes stronger with the LP pump laser. Figure 3(b) presents the calculated ionization probabilities of the excited B^3^Π_g_, a^1^Π_g_ and C^3^Π_u_ states of nitrogen molecules using the ADK theory^26^,^27^. Potential energy curves of these excited states are depicted in inset of Fig. 1 28. In the calculation, the diameter of the focal spot of the probe laser is estimated as ~58 μm if we assume the probe pulse is focused into vacuum, and the pulse duration is 100 fs. As indicated by a blue dashed line, 84% nitrogen molecules in the excited C^3^Π_u_ state will be ionized at the probe energy of 10 μJ. In contrast, nitrogen molecules in the excited B^3^Π_g_ and a^1^Π_g_ states are hardly ionized at such low laser energy. Based on the analysis, the majority of the nitrogen molecules will populate on the excited C^3^Π_u_ state under our experimental conditions. Furthermore, the nonlinear polarizability in the highly excited C^3^Π_u_ state is higher than that in those lower excited states^13^,^14^. Therefore, we expect that the TH signal generated with the CP pump most likely originates from the nitrogen molecules on the excited C^3^Π_u_ state. Nevertheless, to provide an unambiguous answer to this question, some unidentified effects which may also contribute to the THG must be clarified. For example, it is unclear how significantly the vector correlation effects and the difference in the absorption cross sections of different excited states would influence the result, which will be systematically investigated in the future.

**Figure 4:**
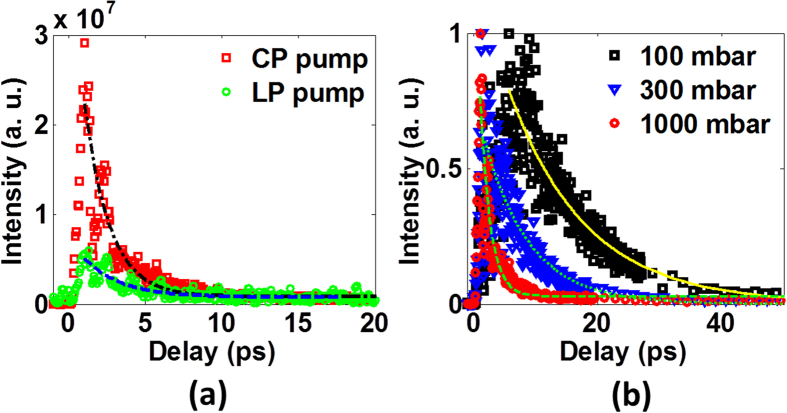
(**a**) The temporal evolution of TH signal in the cases with the CP pump and the LP pump. (**b**) The temporal evolution of TH signal generated with the CP pump at the gas pressures of 100 mbar, 300 mbar and 1000 mbar.

Next, using the TH signal generated with the pump-probe scheme as a probe, we investigate the dynamics of the excited state of nitrogen molecules. As shown in Fig. 4(a), we firstly measured the intensity of TH signal generated with CP and LP pump pulses as a function of the time delay between the pump and the probe at the gas pressure of 1000 mbar. Here, the zero delay was calibrated by the strongest sum frequency signal of the pump and the probe pulses in the nitrogen gas. Clearly, in both cases of the LP pump and the CP pump, the TH signal shows a rapid increase and then reaches its maximum at ~1.3 ps followed by an exponential decay. By exponentially fitting the measured data, the decay lifetimes (defined as the time for the TH intensity to drop to 1/e) are estimated to be ~1.8 ps and ~3 ps for the case of CP pump and the LP pump, respectively. Similar behaviors in two cases show that the observed TH signal has the same origin. As reported by D. Kartashov *et al.*^23^, the longer-wavelength LP laser field can also provide hotter electrons for impact excitation. Therefore, we could also observe TH signal with the LP pump laser at the wavelength of 1420 nm, although it is much weaker than that with the CP pump. Furthermore, we compared the temporal evolution of the TH signal generated with the CP pump at three different gas pressures (i.e., 100 mbar, 300 mbar and 1000 mbar). As shown in Fig. 4(b), with the increase of the gas pressure, the TH signal takes less time to reach its maximum and shows a faster decay, because the collision between the electrons and molecules takes place more frequently at higher molecular densities.

**Figure 5:**
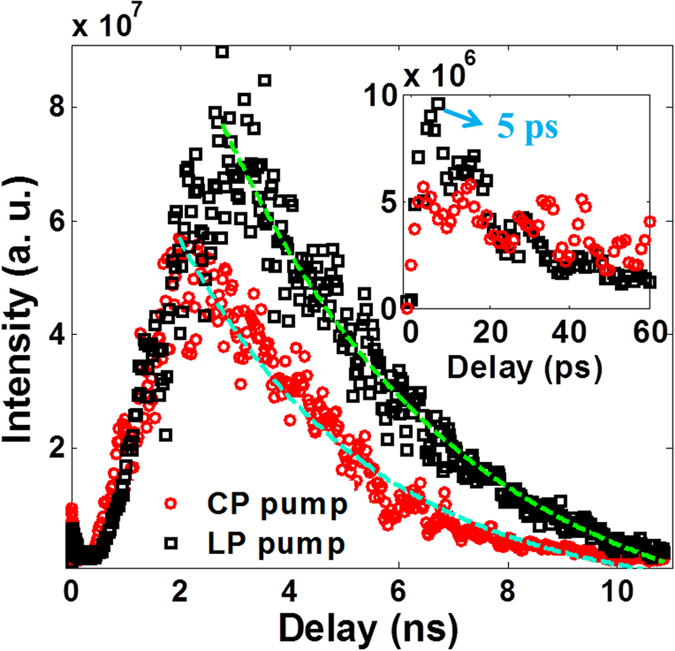
The temporal evolution of TH signal generated in the argon gas under the same experimental conditions asFig. 4(a). The inset shows the time-dependent TH signal in the time window of 0 ~ 60 ps.

To provide a further evidence on the observed generation of TH from the excited molecules, we also performed a comparative investigation in argon gas with the same experimental parameter as Fig. 4(a). As illustrated in Fig. 5, with the increase of time delay between the pump and the probe, the TH signal appears two peaks. For clarity, the inset in Fig. 5 shows the time-dependent TH signal in the time window of 0 ~ 60 ps. Clearly, the first peak reaches its maximum at ~5 ps followed by an exponential decay with the lifetime of ~30 ps. The characteristic time is comparable to the plasma lifetime, and thus we deduce that the TH signal could originate from the plasma effects of the pump pulse. The second peak is about one order of magnitude stronger as compared to the first one, and it appears at the time delay of ~2 ns and ~2.8 ns for the case of CP pump and LP pump, respectively. It is noteworthy that for the main peak, TH signal generated with the LP pump is a little stronger than that generated with CP pump. These features are different from the THG observed in nitrogen molecules. Based on these facts, we suggest that the TH signal after the time delay of ~0.4 ns can be attributed to the formation of the excited metastable state of argon. Previous investigation shows that excited metastable atoms of argon are produced via a two-step process involving three-body collisions Ar^+^ + 2Ar → Ar_2_^+^ + Ar and dissociative recombination Ar_2_^+^ + e → Ar * (4^3^P_2_) + Ar 5. Both two processes are more efficient in the LP laser field due to higher densities of both argon ions and electrons in the LP laser field. From the pump-probe measurements, we can know that the population of argon atoms on the excited metastable state reaches its maximum at 2 ~ 3 ns after the pump pulse. By exponentially fitting the measured data, the decay lifetimes of the excited state are estimated to be ~3.3 ns and ~3.4 ns for the case of the CP pump and LP pump, respectively, which enables the generation of backward nitrogen lasers in the gas mixture of nitrogen and argon^5^,^7^.

In conclusion, we have observed enhanced THG from excited nitrogen molecules and argon atoms. The strong TH signal is attributed to the higher nonlinear polarizability of nitrogen molecules and argon atoms on the excited states. With a pump-probe scheme, we investigate the decay dynamics of excited nitrogen molecules at different gas pressures. Our results provide complementary information other than the temporal dynamics of population inversion in the femtosecond laser induced free-space molecular nitrogen lasers.

## Methods

### Pump-probe setup

The mid-infrared femtosecond laser pulses were provided by an OPA (HE-TOPAS, Light Conversion, Ltd.) pumped by a commercial Ti:sapphire laser system (Legend Elite-Duo, Coherent Inc.). The Ti: sapphire laser system delivered a ~6.3 mJ pulse with a center wavelength of ~800 nm and a pulse duration of ~40 fs at a repetition rate of 1 kHz. The output wavelength from OPA can be continuously tuned in a broad range from 1150 to 2500 nm. In this experiment, a signal beam at the wavelength of 1420 nm was used as the pump and an idler beam at the wavelength of 1820 nm served as the probe. The pulse energy of the pump and the probe was fixed at ~630 μJ and 2 μJ, respectively. The pump was used to prepare the excited states, while the probe was used to generate TH from the molecules on the excited states. A broadband QWP (1100–2000 nm) was inserted into the pump path to change its ellipticity. The probe pulse was set to be linearly polarized. The time delay between the pump and probe pulse was controlled by a motorized translation stage with a temporal resolution of ~16.7 fs. After being combined by a dichroic mirror (DM1), the pump and the probe pulses were collinearly focused into a gas chamber filled with nitrogen or argon by an *f* = 15 cm lens.

### Measurement of TH signal

The generated TH signal after the chamber was first collimated by an *f* = 15 cm lens and then was reflected by another dichroic mirror (DM2) to remove the residual pump and probe lights. Furthermore, a Glan-Taylor (GT) prism was used to minimize supercontinuum white light generated by the intense pump pulse. With the method, we can effectively improve the signal-to-noise ratio of the TH signal. Lastly, the TH signal was recorded by a 1200-grooves/mm grating spectrometer (Andor Shamrock 303i) after being focused on the slit with an *f* = 15 cm lens.

## Additional Information

**How to cite this article**: Xie, H. *et al.* Mid-infrared ultrafast laser pulses induced third harmonic generation in nitrogen molecules on an excited state. *Sci. Rep.* **5**, 16006; doi: 10.1038/srep16006 (2015).
